# Unveiling the mix-up: investigating species and unauthorized tissues in beef-based meat products

**DOI:** 10.1186/s12917-024-04223-4

**Published:** 2024-08-24

**Authors:** Nady Khairy Elbarbary, Wageh S. Darwish, Ahmed Fotouh, Mohamed K. Dandrawy

**Affiliations:** 1 Food Hygiene and Control Department, Faculty of Veterinary Medicine, Aswan University, Aswan, 81528 Egypt; 2 Food Hygiene, Safety, and Technology Department, Faculty of Veterinary Medicine, Zagazig University, Zagazig, 44519 Egypt; 3 Pathology and Clinical Pathology Department, Faculty of Veterinary Medicine, New Valley University, Kharga, Egypt; 4 Food Hygiene and Control Department, Faculty of Veterinary Medicine, South Valley University, Qena, 83522 Egypt

**Keywords:** Meat adulteration, Meat products, Unauthorized tissues, Meat species, PCR

## Abstract

Customers are very concerned about high-quality products whose provenance is healthy. The identification of meat authenticity is a subject of growing concern for a variety of reasons, including religious, economic, legal, and public health. Between March and April of 2023, 150 distinct marketable beef product samples from various retailers in El-Fayoum, Egypt, were gathered. There were 30 samples of each of the following: luncheon, kofta, sausage, burger, and minced meat. Every sample underwent a histological investigation as well as subjected to a standard polymerase chain reaction (PCR) analysis to identify meat types that had not been stated by Egyptian regulations. According to the obtained data, the meat products under scrutiny contained a variety of unauthorized tissues which do not match Egyptian regulations. Furthermore, the PCR results indicated that the chicken, camels, donkeys, and pigs derivatives were detected in 60%, 30%, 16%, and 8% of examined samples, respectively. In conclusion, besides displaying a variety of illegal tissues, the majority of the meat items under examination were tainted with flesh from many species. As a result, it is crucial to regularly inspect these products before they are put on the market to ensure that they comply with the law and don’t mislead customers Furthermore, it is advisable for authorities to implement rigorous oversight of food manufacturing facilities to ensure the production of safe and wholesome meat.

## Introduction

Meat and its products are a significant source of proteins and provide the body with vital amino acids, fatty acids, vitamins, and other elements needed for survival. The demand for higher-quality beef has increased along with improvements in living standards for the world’s population [1]. One of the foods most commonly contaminated in commercial marketplaces is meat, frequently through intentional deception. Using cheaper meat from other animal species to replace more costly or allowed meat, partially or totally, is a common fraud in the meat industry [2]. Many meat products nowadays may contain several species in different proportions mixed together and undetectable by the naked eye or by eating [3]. Most significantly, meat frauds risk the food safety and even threaten public health such as metabolic disorders, allergies and infectious illnesses, because both inedible and edible meat products can sometimes cause allergic responses particularly for sensitized patients [3]. Therefore, food labeling standards must ensure that customers are accurately informed about the types of meat present in food products [4].

Due to halal and kosher regulations, as well as the preferences of other religious affiliations, failing to declare the animal species contained in food violates consumer rights and trust. It poses a serious risk to religious groups [5]. Precise identification of animal species in meat products is essential for safeguarding human health as well as promoting equitable commerce among meat producers [2]. In numerous countries, food adulteration is a serious concern due to the potential risks to public health and potential financial losses. Food adulteration makes food seem better or weigh more, but it lowers the meal’s quality [6]. However, Egyptian Organization Standards (EOS) stated that beef-based products should be made from the meat of Halal animals and should be written clearly on the label; As well as beef-based products should be free of cartilage, bones, tendon, ligaments, visible blood vessels, blood clots mucous membranes, brain, stomach, and intestines (No. 1694/2005, 1973/2005, 1972/2005, 1688/2005, and 1114/2005). Regular auditing procedures and analyses to determine the animal species present are essential for preventing adulteration and fraud in these items [2]. The food industry’s expansion and customer safety depend on creating quick and effective techniques for detecting adulterated meat [7].

Despite the significant effort invested in the review and updating of outdated laws and regulations in Egypt, they are still not aligned with the standards and principles of Codex and other international organizations. There are still many things to be done to assure food safety from all sources, such as infrastructural development, legislative changes, public awareness, and so on. In today’s complex food supply chain, ensuring the safety and integrity of food products is of utmost importance. With the growing concern over intentional adulteration and food fraud, food companies must implement robust management systems to mitigate risks and safeguard consumers. By implementing Threat Assessment Critical Control Point (TACCP), A food company demonstrates its commitment to ensuring food safety and integrity. Through proactive threat assessment, risk mitigation, and control measures, the company mitigates the risk of intentional adulteration and protects consumers from potential harm. As threats continue to evolve, ongoing vigilance and adaptation are essential to maintaining the effectiveness of TACCP in safeguarding the food supply chain [8].

Most analytical techniques created to confirm meat species labeling claims and identify adulteration include assays based on proteins, metabolites, or nucleic acids [9]. Histological analysis could look into certain additional protein kinds, including the proteins found in plant and animal tissues. According to numerous research [2, 6, 10, 11], histological exams are the recommended methods of quality control since they may identify fraud in food and meat products. Moreover, DNA-based techniques such as the polymerase chain reaction (PCR), a molecular biological method used in food authentication, are considered very accurate and quick [9]. This study aimed to look into possible adulteration in meat products produced locally and trademarked in El-Fayoum City, Egypt, that are marketed as 100% beef by using histology and PCR techniques.

## Materials and methods

### Collection and sample preparation

In March and April of 2023, a total of 150 samples of various commercial packaged beef products, including 30 each of minced beef, kofta, sausage, burgers, and luncheon, were randomly gathered from local and high-end supermarkets in various regions of El-Fayoum, Egypt. After 50 g of the product sample was packed and labeled, each sample was shipped in an icebox container to the Aswan University Faculty of Veterinary Medicine’s Central Lab and stored at 4 °C for examination [12].

### Application of the standard histology approach to identify animal and herbal tissues in beef products

Briefly, the specimens were obtained, cut into 1 × 1 × 0.5 cm sections, and preserved for 72 h in 10% neutral buffered formalin. Samples were processed by dehydration in ascending serial grades of ethanol (70%, 80%, 90%, and absolute), cleared in Xylene, sample impregnation, and embedded in Paraplast tissue embedding media. Serial 5µn thin tissue sections were cut by rotatory microtome (SLEE, CUT 4062, Germany) and fixed to glass slides. Tissue sections were stained by Harris Hematoxylin and Eosin (H&E) as a general tissue examination staining method. The slides were observed under a microscope with a digital camera (Optika, TCB5, ver. 2.1, Italy) to detect additive tissues [13].

### Detection of meat species adulteration by PCR

Using specialized primers that target the (*cytb*) gene of various meat species, including chicken, camel, pig, and donkey, animal derivatives can be identified in meat products using a conventional PCR technique. The samples were analyzed using the PCR approach to recognize meat species adulteration [14]. Furthermore, this investigation section used the DNA of four distinct meat samples from identified species, including chicken, camel, pork, and donkey, as a positive control.

### DNA extraction

Following the directions of the *Quick*-gDNA™ MiniPrep kit (Catalog No. D3024, Zymoresearch, USA). After DNA extraction, NanoDrop 1000 (Germany) UV photometry was utilized to ascertain the integrity and concentration of DNA solutions.

### Oligonucleotide primers

Four sets of oligonucleotide primers for targeted (*cyt b*) gene of different animals (chicken, camel, pork, and donkey) synthesized by Willowfort Company (United Kingdom) were incorporated in the present study for the detection of chicken, camel, pork, and donkey species in beef product samples (Table 1).

**Table 1:** The specific sequences and amplified products of oligonucleotide primers

Primer	Gene	Primer sequence 5’-3’	bp	Reference
Chicken	*cyt b*	CTCCCATAGACAGCTCC CCCCAAAAAGAGAAGGAA	442	Bellis et al. [15]
Camel	*cyt b*	AGCCTTCTCTTCAGTCGCACAC GCCCATGAAAGCTGTTGCT	208	Chen et al. [16]
Porcine	*cyt b*	GCCTAAATCTCCCCTCAATGGTA TGAAAGAGGCAAATAGATTTTCG	212	Mane et al. [17]
Donkey	*cyt b*	ATCCTACTAACTATAGCCGTGCTA CAGTGTTGGGTTGTACACTAAGATG	439	

### DNA amplification and examining the outputs

The PCR amplification was achieved in a 50 µl reaction volume using 25 µl *COSMO* PCR RED Master Mix (Code No. W1020300X, Willowfort, United Kingdom), 1 µl of each primer, 1 µl extracted DNA, and 22 µl of Nuclease free water. Through electrophoresis, PCR products were distributed across a 1.5% agarose gel (Applichem GmbH, Germany) in 1x TBE buffer at ambient temperature. Gradients of 5 V/cm were implemented, and 15 µl of the products were loaded into each gel slot. The resultant gel was subjected to staining with 0.5 µg/ml Ethidium bromide, followed by visualization via UV transilluminator and digital camera photography [18].

### Statistical analysis

For histological results, the mean ± standard error was used to represent the ratio of the area surface of the additive tissues to the total area surface for each photograph. The *t-test* was used to determine significant differences between the real and the estimated percentages. The significant differences between the different sources of adulterated samples were tested using Chi Square and a one-way ANOVA (SPSS 16.0 statistical software, 2001). A value of *p*˂0.05 was considered significant.

## Results

### Detection of unauthorized tissues based on histological examination

The results showed that all samples contained muscular tissue in percentage of 100%, 80.27%, 80%, 74.33%, and 40%, respectively, for minced meat, kofta, sausage, burger, and luncheon (Table 2). Furthermore, the examined meat samples had adipose tissue, fascia/tendon, bone/cartilage, blood vessels, lung tissue, plant tissue, and glandular tissue with a mean total meat adulteration (%) of 18.24 ± 2.12, 19.05 ± 2.08, 18.22 ± 2.51, 17.55 ± 2.69, and 10.35 ± 1.20, respectively for minced meat, kofta, sausage, burger, and luncheon based on histological examination (Table 3) and the differences in the percentage of the detected tissues were considered not significant at (*p*>0.05).

**Table 2:** Detected tissues (%) in beef products based on histological examination

Detected tissues	Minced beef	Kofta	Sausage	Burger	Luncheon
Muscular tissue	100	80.27	80	74.33	40
Adipose tissue	82	54.27	72.27	60.82	33
Fascia / Tendon	70.13	86.22	75	88.25	36.42
Bone / Cartilage	35.63	61	70.33	60	33
Blood vessels	55.77	34.44	30.33	55.37	0
Lung tissue	18.75	30.1	5	0	0
Glandular tissue	17.22	25.22	28	5.55	42
Plant tissue	62	88	76.67	90	55

**Table 3:** Mean value of beef product adulteration percentage based on histological examination

Product	No.	Mean ± S.E
Minced beef	30	18.24 ± 2.12^a^
Kofta	30	19.05 ± 2.08^a^
Sausage	30	18.22 ± 2.51^a^
Burger	30	17.55 ± 2.69^a^
Luncheon	30	10.35 ± 1.20^b^

**Fig. 1:**
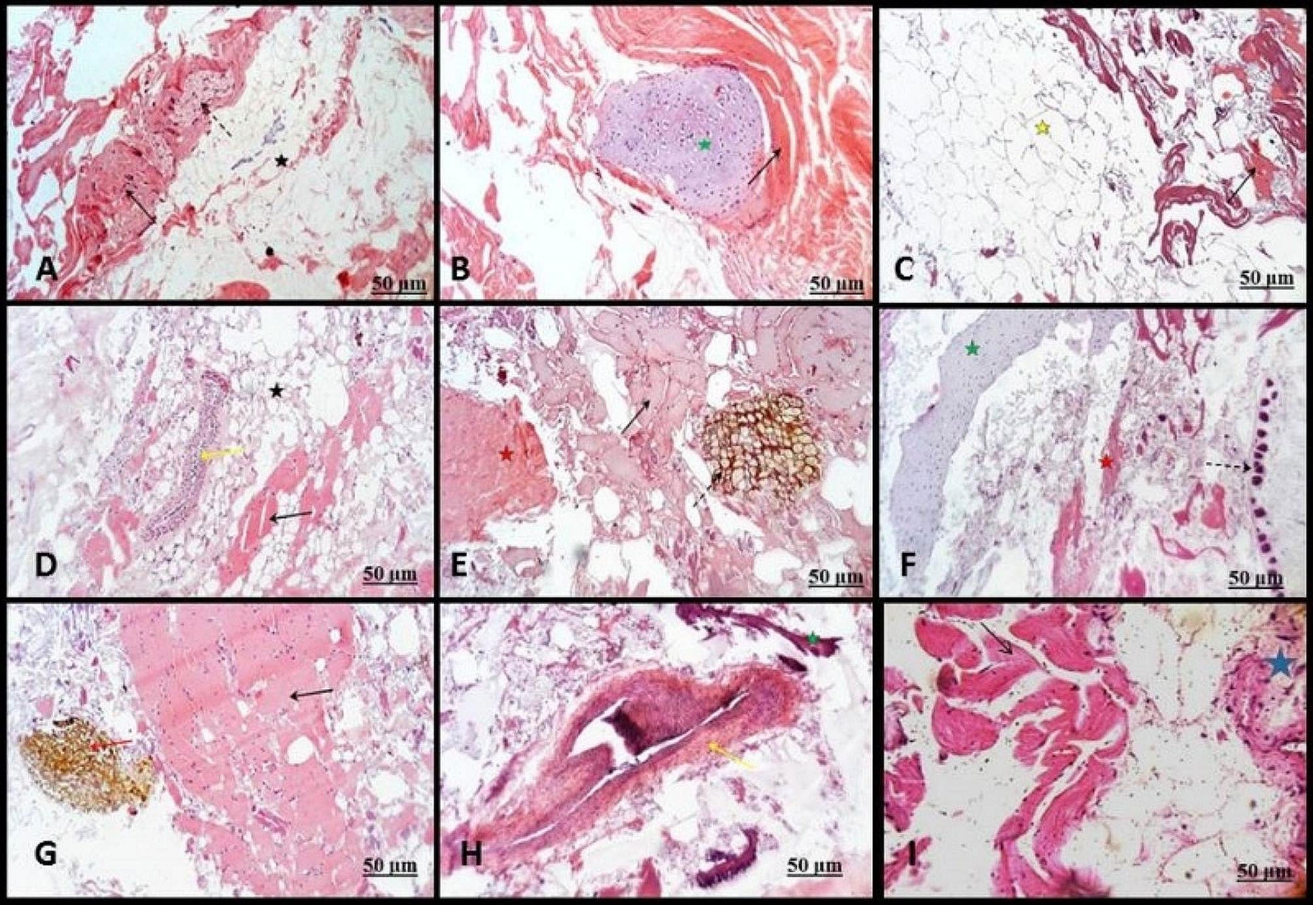
Paraffin sections showing the detected tissues in minced beef (**A**, **B**, and **C**), sausage (**D**, **E**, and **F**), and burger (**G**, **H**, and **I**) products stained with H&E. (**A**): The meat samples contained muscular tissue (black arrow), plant root (dashed arrow), and a part of the adipose tissue contained white fat cells (black star). (**B**): Muscular tissue (black arrow), Bone/cartilaginous tissue (green star). (**C**): Muscular tissue (black arrow) and lung tissue (yellow star). (**D**): Muscular tissue (black arrow), blood vessels (yellow arrow), and adipose tissue (black star). (**E**): Muscular tissue (black arrow), plant tissue (dashed arrow), and fascia /tendons (red star). (**F**): A part of a plant stem (dashed arrow), bone/cartilaginous tissue (green star), and fascia /tendons (red star). (**G**): Muscular tissue (black arrow), Spices (red arrow). (**H**): Blood vessel (yellow arrow) and bone/cartilaginous tissue (green star). (**I**): Muscular tissue (black arrow) and nerve cells (blue star)

**Fig. 2:**
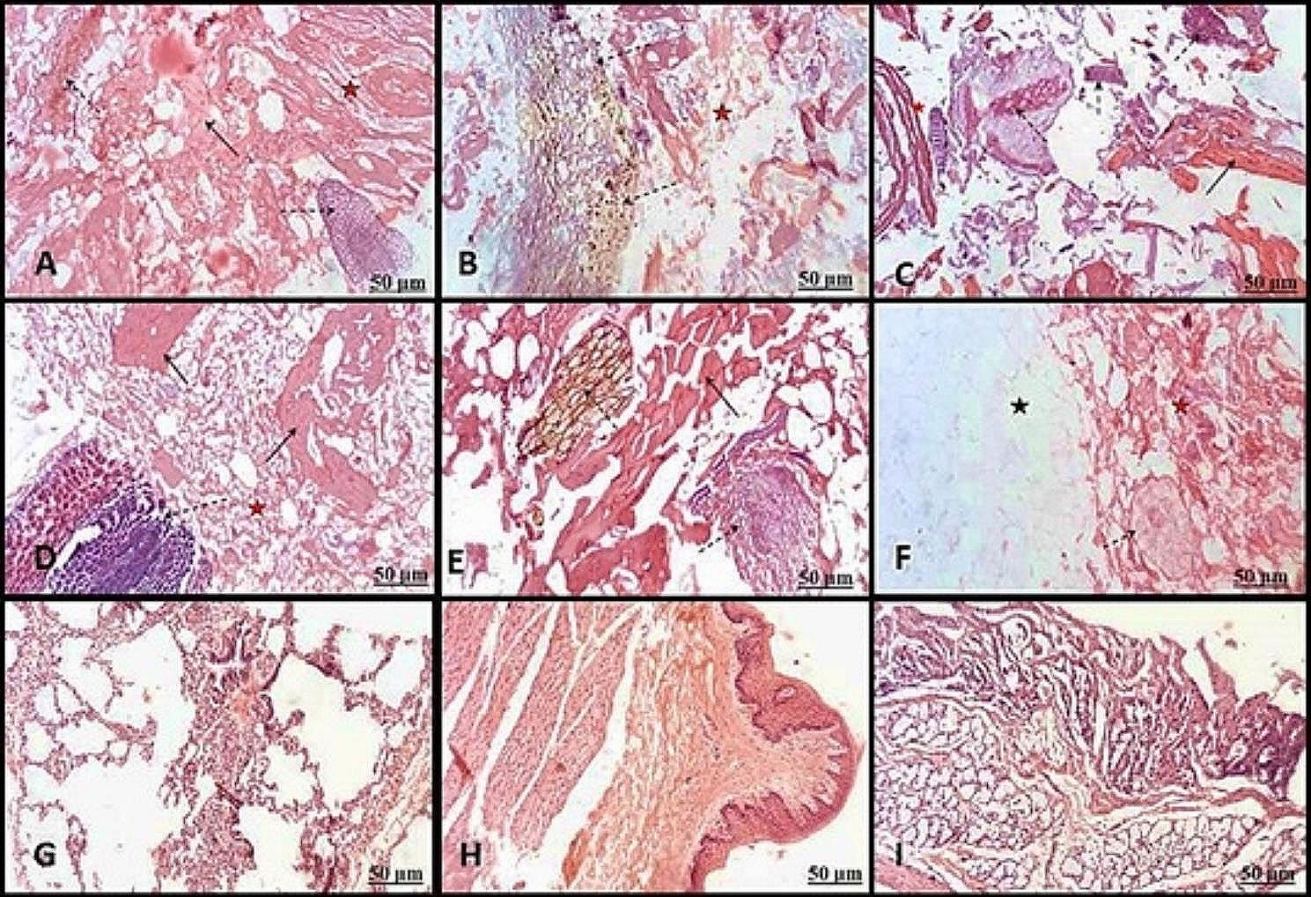
Paraffin sections showing the detected tissues in luncheon (**A**, **B**, **C**, **D**) kofta (**E**, **F**, **G**, **H**, **I**) products stained with H&E. (**A**): The meat samples contained muscular tissue (black arrow), plant root (dashed arrow), and fascia /tendons (red star). (**B**): plant root (dashed arrow) and fascia /tendons (red star). (**C**): muscular tissue (black arrow), plant root (dashed arrow), and fascia /tendons (red star). (**D**): muscular tissue (black arrow), plant leaf (dashed arrow), and fascia /tendons (red star). (**E**): muscular tissue (black arrow), plant root and leaf (dashed arrow), and fascia /tendons (red star). (**F**): a part of the adipose tissue contained white fat cells, A part of the plant stem (dashed arrow), and fascia /tendons (red star). (**G**): lung tissue was identified by alveoli and basophilic cartilage remnants. (**H**): Ruminal tissue characterized by keratinized stratified squamous epithelium. (**I**): Intestinal tissue was identified by its villi, columnar epithelium, and smooth muscular coat

Figures 1 and 2 show the histological inspection of the detected tissues in examined products, including muscular tissue (Figs. 1A, B, C, D and E and 2A, C, D and E), plant leaves were identified by cuticle, epidermis, mesophyll, parenchyma cells, and vascular tissue (Figs. 1A, E and I and 2A and D), a part of plant stem (Fig. 1F), plant root (Fig. 2B, C and D), a part of the adipose tissue contained white fat cells (Figs. 1A and D and 2F), bone/cartilaginous tissue with characteristic trabeculae of spongy bone separated by bone marrow (Fig. 1B, F and H), lung tissue was recognized by alveoli, basophilic cartilage remnants (Figs. 1C and 2G), Vascular tissue was characterized by the elastic and muscular artery. The regularly arranged elastic fibers recognized the elastic artery. In contrast, the muscular artery was known by the muscular layer in the tunica media (Fig. 1D and H), fascia /tendons were characterized by regularly arranged dense collagen fibers and tendons characteristic by bundles of parallel collagen fibers (Figs. 1E and I and 2A, B and E), Spices (Fig. 1F), nerve cells (Fig. 1I), ruminal tissue characterized by keratinized stratified squamous epithelium and honeycomb-shaped reticular folds (Fig. 2H) and intestinal tissue (hollow or tubular organs) was identified by their villi, columnar epithelium and the smooth muscular coat; inner circular and outer longitudinal smooth muscle fibers (Fig. 2I).

### Detection of adulteration with different meat species by PCR technique

**Fig. 3:**
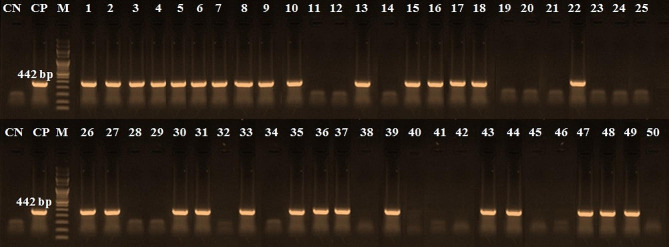
PCR amplification pattern for chicken derivatives detection at 442 bp in examined beef products. CN: control negative, CP: control positive, M = Marker (100 bp), Lane 1 to Lane 10: minced beef samples, Lane 11 to Lane 20: kofta samples, Lane 21 to Lane 30: sausage samples, Lane 31 to Lane 40: burger samples, and Lane 41 to Lane 50: luncheon samples

**Fig. 4:**
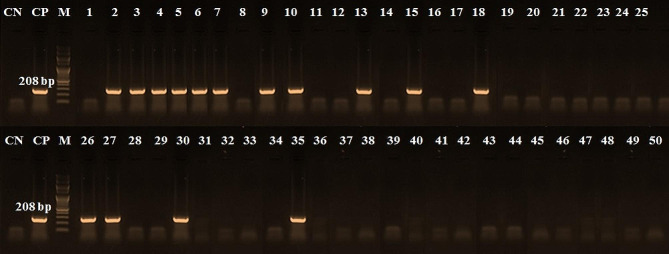
PCR amplification pattern for camel derivatives detection at 208 bp in examined beef products. CN: control negative, CP: control positive, M = Marker (100 bp), Lane 1 to Lane 10: minced beef samples, Lane 11 to Lane 20: kofta samples, Lane 21 to Lane 30: sausage samples, Lane 31 to Lane 40: burger samples, and Lane 41 to Lane 50: luncheon samples

**Fig. 5:**
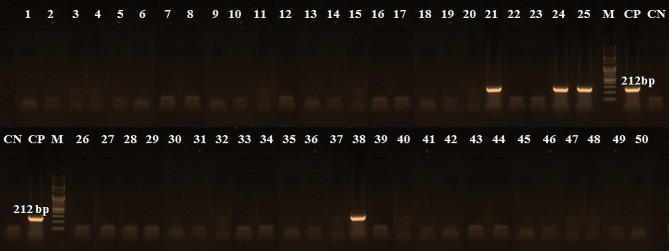
PCR amplification pattern for pig derivatives detection at 212 bp in examined beef products. CN: control negative, CP: control positive, M = Marker (100 bp), Lane 1 to Lane 10: minced beef samples, Lane 11 to Lane 20: kofta samples, Lane 21 to Lane 30: sausage samples, Lane 31 to Lane 40: burger samples, and Lane 41 to Lane 50: luncheon samples

**Fig. 6:**
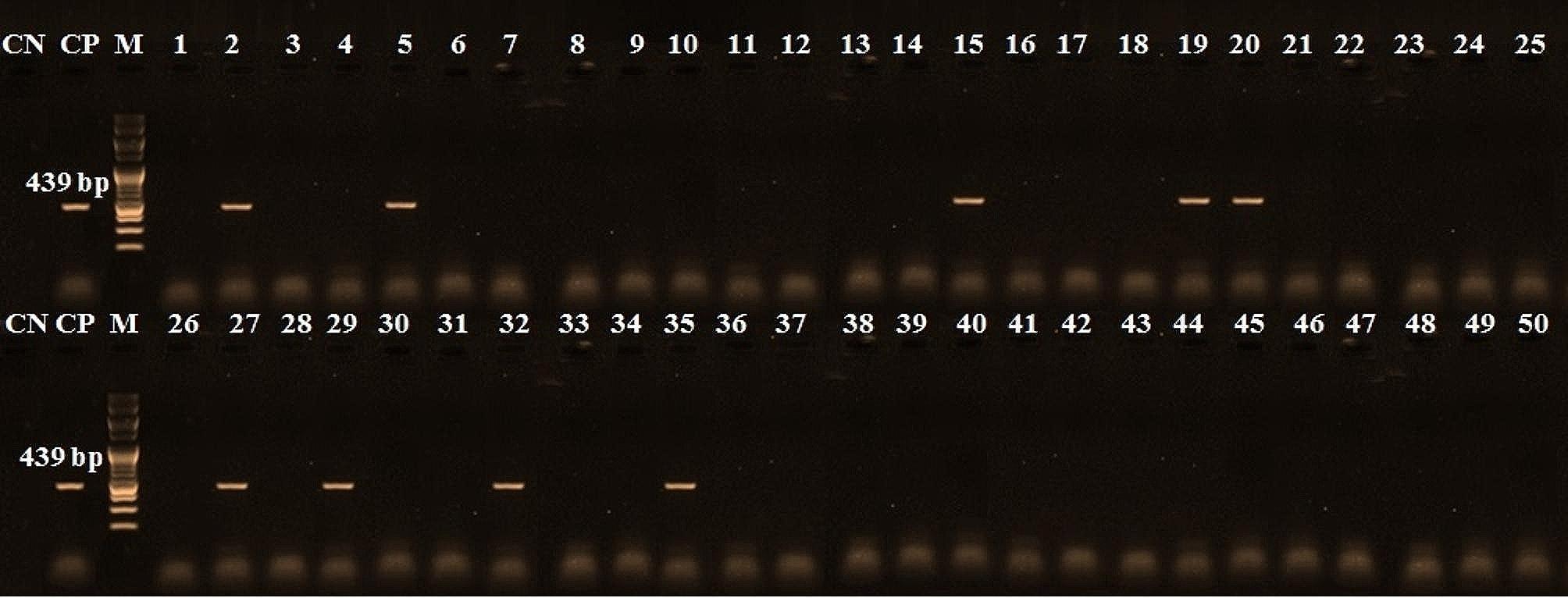
PCR amplification pattern for donkey derivatives detection at 439 bp in examined beef products. CN: control negative, CP: control positive, M = Marker (100 bp), Lane 1 to Lane 10: minced beef samples, Lane 11 to Lane 20: kofta samples, Lane 21 to Lane 30: sausage samples, Lane 31 to Lane 40: burger samples, and Lane 41 to Lane 50: luncheon samples

Table 4 shows the incidence of detected species in the examined beef products by PCR. These results indicated that the chicken derivatives were detected in 60% of examined samples (Table 4), which occur in all minced meat samples, 60% of burgers, and 50% of each kofta and luncheon samples while sausage has 40% chicken materials (Fig. 3). Furthermore, 30% of examined samples have camel derivatives present in 80% of minced meat, 30% of each kofta, sausage, and 10% of burger samples. In contrast, luncheon was free from camel derivatives (Fig. 4). Additionally, 8% of the samples contained pig genetic material, minced meat, kofta and luncheon samples were free from pig derivatives but present only 30% and 10% of sausage and burger samples (Fig. 5). On the other hand, donkey genetic material was detected in 18% of the samples where 20% of each minced meat, sausage, and burger samples and 30% of kofta samples had donkey derivatives while luncheon samples free from donkey derivatives (Fig. 6).

**Table 4:** Total adulteration (%) and the detected species in the examined beef products (*n* = 10 of each)

Detected species	Adulteration rate		Minced beef		Kofta		Sausage		Burger		Luncheon	
	No.	%	No.	%	No.	%	No.	%	No.	%	No.	%
Beef	50	100	10	100	10	100	10	100	10	100	10	100
Chicken	30	60	10	100	5	50	4	40	6	60	5	50
Camel	15	30	8	80	3	30	3	30	1	10	0	0
Pig	4	8	0	0	0	0	3	30	1	10	0	0
Donkey	9	18	2	20	3	30	2	20	2	20	0	0

## Discussion

### Detection of unauthorized tissues based on histological examination

In food regulatory control, determining the authenticity of the meat in meat products is critical for avoiding fraudulent substitution of more expensive commercial meat species with inferior, cheaper, or unwanted substitutes, detecting the attendance of undeclared species, replacing animal meat with plant proteins, accurately labeling food, evaluating food composition, and giving consumers the information they need to ensure food safety [19].

The meat products investigated in this study are widespread in Egypt, as presented by the increased manufacturing and selling capacity over time [10]. Accordingly, this investigation assessed the quality of these widely consumed meat products by employing a histology technique to determine the occurrence of forbidden tissues in various beef products prepared to the Egyptian public. For quality control of such meals, histological techniques can be an easy, quick, affordable, conclusive, and definitive tool to identify unapproved tissues [20]. The results of the histological analysis in this study show that all samples had muscle tissue in varying percentages as well as included various types of unapproved tissues, including blood vessels, fascia/tendon, bone/cartilage, lung tissue, plant tissue, and glandular tissue. Therefore there were some products not in agreement with EOS in the current investigation as EOS stated that beef-based products should be free of cartilage, bones, tendon, ligaments, visible blood vessels, blood clots mucous membranes, brain, stomach, and intestines (No. 1694/2005, 1973/2005, 1972/2005, 1688/2005, and 1114/2005).

The present findings were consistent with those of Javad et al. [21] in Tehran, Iran, reported that 100% of the slides showed the striated skeletal muscles accompanying glandular tissues. Additionally, Mokhtar et al. [11] in Assiut, Egypt, found that a variety of animal tissues, including abundant adipose tissue (50%), bone (7.1%), heart muscles (3.6%), smooth muscle fibers of internal organs (25%), lung tissues (25%), nerve cells (3.6%), fascia (3.6%), connective tissue (10.7%), blood vessels (7.1%), and plant tissues (14.3%), were mixed in with the minced meat. Histological analyses have been the favored method of quality control as they have been shown in various studies to identify fraud in meat and food products [6, 10]. According to the results of the current investigation, histological examination revealed the presence of several different tissue types in addition to skeletal muscle, such as connective tissue fibers, lung, fascia/tendon, blood vessels, adipose tissue, cartilage (white and hyaline fibrocartilage), spongy bone, plant materials, and glandular tissue (Figs. 1 and 2).

The present study’s findings are consistent with other research that assessed unapproved tissues in meat products in Egypt [6, 22] and Iran [10]. Furthermore, Malakauskiene et al. [23] reported that while cartilage and bone were absent from the samples under examination, striated muscle, adipose and connective tissue, blood vessels, glandular tissue, and nerves were present in the samples from Kaunas, Lithuania. To determine the amount of unapproved animal and herbal content in minced beef meat, Sadeghinezhad et al. [13], in Tehran, Iran, focused on the qualitative and quantitative correctness of histological examinations where 5%, 10%, 15%, and 20% of unauthorized tissues were composed. Furthermore, the current investigation demonstrated that the burger had a nerve trunk (Fig. 1I), supporting the notion that prohibited tissues, like the brain and spinal cord, may harbor infectious pathogens that pose a risk to consumers [24].

The investigation of alveolar tissues of the lung in minced meat and Kofta (Figs. 1C and 2G) was consistent with the study of [6, 25] in Egypt and Turkey. The acquired results, consistent with the findings of Abdel-Maguid et al. [6] in Assiut City, Egypt, demonstrated the presence of immature or growing long bone in the minced meat and sausage samples (Fig. 1B and F). As bone fragments are not typically found in meat products, their occurrence is unusual and can indicate a problem with processed meat [26], which impacts the product quality. The presence of bone tissue in the studied samples may indicate that these meat products were contaminated with dead fetuses, as fetal material is not permitted in meat due to its sloppy appearance and poor excellence. Furthermore, it is a consequence of abortions, which may be induced by pathogens and have the potential to significantly contaminate food supplies and infect customers [22]. The investigation unveiled the occurrence of degenerative muscle in every category of specimens.

Consumers should know processed foods and the distinctions between the first and second types of skeletal muscle (light fiber, slow contracting, and slow contracting, respectively). This information is significant since the recognition of meat softness depends on the ratio of different fiber types based on the quantity of light and dark fibers [6]. All morphological traits and plant structures that lower meat quality and compromise food safety as allergens, as reported by [11, 22] in Egypt, suggest that plant additives or spices are present in meat products, as well as food remnants from animals’ stomachs whose meat muscles are used to prepare meat products. According to [22] in Egypt and [27] in İstanbul, the high concentration of fascia (dense fibrous connective tissue) and cartilage (either hyaline or white fibrocartilage) in an extreme quantity in all samples suggests that their addition lowers the excellence and nutritious value of the meat used.

From an ethical and theological perspective on human health and an economic one, Identifying animal tissues in meat products is critical to consuming healthy foods free of illegal competition and undesired adulteration. To protect customers from fraudulent and counterfeit meat substitution techniques, histological methods could be used in routine examinations to ensure the authenticity and quality of all sorts and forms of meat-based products. The outcome discrepancy may be attributed to the random selection of sections from the utilized organ and the nature of the products being analyzed.

### Detection of adulteration with different meat species by PCR

Consumers around the world are increasingly worried about the adulteration of meat products. Therefore, regarding food regulation and consumer protection, the sort of meat used to make the meat product is an extremely important consideration. Conversely, the discernment of meat varieties within diverse beef products is critical, particularly in Islamic nations where Halal meat is the only option for consumption [28]. As a result, this investigation evaluated the quality of these widely consumed meat products by employing a PCR approach to determine the species content in different beef products made available to the Egyptian public. Food safety in Egypt is being challenged nowadays by the global dimensions of food supply chains. The features of the Food Safety System in Egypt are based on the results of the diagnostic study and the organizational structure of food safety is multi-sectorial within the model called “multiple bodies system,” and there is no identifiable single strategic plan for the food safety system. Besides, there were several bodies involved in official food control, whose fields of action and organization structures are clearly defined [29]. However, the Egyptian Food Codex forbids the addition of poultry and other species’ meat to products branded 100% beef.

Additionally, the current research has shown that the PCR method is useful for quantitatively identifying meat species. According to Özlü et al. [2], this enables the faster, simpler, and more dependable identification of foods from animals that humans do not normally eat. Furthermore, the occurrence of the discovered species in the beef products under examination was displayed in Table 4. The amplification was unaffected by processing or additives, and the presence of non-target DNA had no discernible impact on detecting the intended DNA (Figs. 3, 4, 5 and 6). Other researchers have come to the same conclusion in Egypt [30, 31] and [2, 9] in Türkiye. Different undeclared species, including chicken (100%), camel (80%), and donkey (20%) derivatives, were detected in the minced beef analyzed in this investigation using PCR. Chicken and camel were the most frequently adulterated species, followed by donkey and pig.

The observed outcomes could be explained by the greater difficulty in visually detecting intentional substitution with undeclared species in these products, as opposed to fresh and intact meat [30]. There may be an economic rationale for adding chicken to beef products because the main cause of adulteration with chicken species is likely their lower cost when compared to beef. According to [32], the occurrence of chicken species may be explained by the enormous amounts of skin, frames, legs, and necks used due to the dramatic rise in poultry production and manufacturing. This shift in consumer consumption from whole chicken to cuts and fillets is also connected to the existence of chicken species. Furthermore, carcasses and other waste items from chickens, known as trimmings, including fat, connective tissues, blood vessels, cartilage, and even small bits of bone, may be combined with meat and utilized as adulterants [33].

According to [34, 35], donkey meat was found in 40% and 56% of the minced beef tested in Egypt. El-Sheikh et al. [30] found that 4% and 5% of minced beef contained pig and donkey meats, respectively, in samples from various markets in Sharkia Governorate, Egypt. Because camel meat is inexpensive and readily available, it is frequently adulterated with other animal species in minced beef products in various Middle Eastern countries, especially Egypt [36]. EOS stated that beef-based products should be made from the meat of Halal animals and should be written clearly on the label; furthermore, donkey meat and pork are forbidden to be including (No. 1694/2005, 1973/2005, 1972/2005, 1688/2005, and 1114/2005).

The outcomes of this study revealed that the kofta samples were contaminated with 50% derivatives of chicken, 30% derivatives of camel, and 30% derivatives of donkey meat. These findings were consistent with those of Hamouda et al. [37] in Egypt while Cetin et al. [38] in Istanbul discovered that 5.6% of the samples were tainted with chicken. Additionally, 90.9% of the meatball samples had impurities, according to Yosef et al. [39]. There are numerous potential causes for the adulteration of livestock products. Reducing production costs is one of the goals to cut costs; adulterators may mix nonmeat substances or less expensive meats with minced meat. Another justification is to mislead customers. Adulterators could pose as selling more expensive meat varieties, including beef, when selling contaminated meat products [40].

The researchers claim that changes in the reaction concentration and adjustments to the temperature and time parameters for each PCR step can affect the outcome [30]. Accordingly, the feature of primers is crucial for accurate authentication of meat species. Target primers showed more stringent specificity and shared similar melting temperature to that of other targets ensuring to anneal with target templates under the same set of PCR conditions [41]. As reported, even a single base pair that mismatches at the 3’ end of the primers with target DNA might interfere with the efficiency of PCR amplification [42]. Furthermore, the specificity of target primers was confirmed based on species-specific amplification of PCR assays.

Moreover, the derivatives of sausage include chicken (40%), camel (30%), pig (30%), and donkey (20%). Similar findings to those of Ahmed et al. [43] showed that 10% and 50% of the samples collected from Ismailia, Egypt, were devoid of pork but contaminated with species of chicken and donkey. In contrast, Yosef et al. [39] found that the contamination rate with pork was 6.6% and 5.5%, respectively, but no donkey species were detected in any of the samples. However, Özlü et al. [2] in Türkiye, verified that the sausage samples under examination were devoid of pig, camel, and donkey meat. The current study’s analysis of the burger indicated that it comprised 60% poultry derivatives, 10% camel derivatives, 10% pig derivatives, and 20% donkey derivatives. Compared to prior studies in Ismailia, Egypt [43] found that 100% and 30% of the burger samples were adulterated with chicken and donkey derivatives, respectively. Additionally, Cawthorn et al. [44] revealed that 40% of the samples from South Africa were adulterated with chicken and 30% with pork. Another significant issue of ethics, regulation, and health was identifying undeclared equine species in a single sample marketed as beef. Customers find donkey meat ugly and terrible to eat; hence, any presence of it is prohibited.

The consistent incorporation of prohibited species, including donkeys and pork, into a variety of products, has alarmed Muslim consumers regarding the ability of merchants to certify that a product is halal [45]. However, the findings indicated that while all the luncheons tested were devoid of camel, pig, and donkey derivatives, 50% of the samples included chicken. The outcomes corroborated the finding by Yosef et al. [39] that no donkey derivatives existed in any of the samples. On the other hand, [35, 43, 46] discovered that 10%, 24%, and 6.6% of the samples collected from Egypt were tainted with donkey meat, whereas Ahmed et al. [43] reported that 70% and 10% of the samples under examination included derivatives of chicken and donkey.

In this research, a sizable fraction of the meat product samples contained undeclared species, indicating that adulteration was probably done on purpose. The results mentioned above offer an intriguing illustration of how certain meat producers can easily profit financially from flaws or ambiguities in local regulations and how adulteration of meat products with different undeclared species has become a common issue in the markets. Therefore, for the sake of the intended consumer base, quick and practical testing by the food safety authority is advised to implement stricter restrictions on industrial beef products.

## Conclusion

Authentication of animal species origin in food is critical, not only for the avoidance of fraud but also for human health and religious issues. Based on the current findings, it is possible to deduce that the meat products under scrutiny contained a variety of unauthorized tissues that do not match Egyptian regulations as well as included one or more species other than beef, which defies the label’s stated information. This highlights the need for the food safety authority to augment routine food safety testing with food defense principles stated by Egyptian regulations. In addition, the histology and PCR procedures are quick and efficient ways to identify unapproved tissues and species, offering significant advantages for consumer rights, food safety, and avoiding unfair competition.

## Data Availability

No datasets were generated or analysed during the current study.

## Declarations

### Guidelines

All methods were carried out in accordance with relevant guidelines and regulations.
